# Asymmetric Bidirectional Transcription from the FSHD-Causing D4Z4 Array Modulates DUX4 Production

**DOI:** 10.1371/journal.pone.0035532

**Published:** 2012-04-20

**Authors:** Gregory J. Block, Lisa M. Petek, Divya Narayanan, Amanda M. Amell, James M. Moore, Natalia A. Rabaia, Ashlee Tyler, Silvere M. van der Maarel, Rabi Tawil, Galina N. Filippova, Daniel G. Miller

**Affiliations:** 1 University of Washington, Department of Pediatrics. Seattle, Washington, United States of America; 2 Fred Hutchinson Cancer Research Center, Human Biology Division, Seattle, Washington, United States of America; 3 University of Rochester Medical Center, Rochester, New York, United States of America; 4 Leiden University Medical Center, Leiden, Netherlands; Florida State University, United States of America

## Abstract

Facioscapulohumeral Disease (FSHD) is a dominantly inherited progressive myopathy associated with aberrant production of the transcription factor, Double Homeobox Protein 4 (DUX4). The expression of *DUX4* depends on an open chromatin conformation of the D4Z4 macrosatellite array and a specific haplotype on chromosome 4. Even when these requirements are met, DUX4 transcripts and protein are only detectable in a subset of cells indicating that additional constraints govern DUX4 production. Since the direction of transcription, along with the production of non-coding antisense transcripts is an important regulatory feature of other macrosatellite repeats, we developed constructs that contain the non-coding region of a single D4Z4 unit flanked by genes that report transcriptional activity in the sense and antisense directions. We found that D4Z4 contains two promoters that initiate sense and antisense transcription within the array, and that antisense transcription predominates. Transcriptional start sites for the antisense transcripts, as well as D4Z4 regions that regulate the balance of sense and antisense transcripts were identified. We show that the choice of transcriptional direction is reversible but not mutually exclusive, since sense and antisense reporter activity was often present in the same cell and simultaneously upregulated during myotube formation. Similarly, levels of endogenous sense and antisense D4Z4 transcripts were upregulated in FSHD myotubes. These studies offer insight into the autonomous distribution of muscle weakness that is characteristic of FSHD.

## Introduction

Facioscapulohumeral Muscular Dystrophy (FSHD) is thought to be caused by aberrant production of a protein called Double Homeobox 4 (DUX4) [1], [2], [3], [4], [5]. *DUX4* is part of a macrosatellite repeat arranged as a head-to-tail array of similar but not identical units called D4Z4 present up to 200 times on human chromosomes 4 and 10 [1]. A critical number of 10 units appears to be required for establishing and maintaining the array as heterochromatin resulting in transcriptional silencing of *DUX4* [6], [7], [8]. The D4Z4 array is assembled as euchromatin when the D4Z4 repeat number is truncated to less than 10 units. This chromatin conformation, in combination with chromosome 4-specific polymorphisms, results in *DUX4* transcription and complete processing of protein coding transcripts [2], [5], [9], [10], [11]. DUX4 is toxic to multiple cell types and tissues, including skeletal muscle [12], [13], [14], [15], [16], suggesting a mechanism for the muscle dystrophy and weakness present in people with FSHD.

The design of FSHD treatment strategies requires a thorough understanding of the regulation of *DUX4* expression. Associations of D4Z4 with other distant sequences have been described and suggest that D4Z4 sequences participate in long-range chromosomal interactions that mediate transcription of chromosome 4 genes centromeric to D4Z4 [17]. A CTCF and type-A lamin dependent insulator prevents silencing of adjacent reporter genes [18], and a potent enhancer that binds Kruppel-like factor 15 [19] has been described within D4Z4 that could activate distant genes in assays of chromosome looping [17]. Although these interactions are likely important for FSHD pathogenesis, this study differs from the previous reports by demonstrating how sequences within D4Z4 affect cis-regulation of *DUX4* transcription. In particular we focus on the fact that packaging of the D4Z4 array as euchromatin alone is not sufficient to produce functional *DUX4* transcripts since at any particular time full-length transcripts and protein are restricted to a subset of nuclei within myotubes even when permissive haplotypes are present [4].

Transcriptional mechanisms governing sporadic *DUX4* expression are likely to be important for understanding disease pathogenesis and treating FSHD. The asymmetric distribution of muscle weakness seen in patients suggests that conditions can be present in some muscles that result in relative sparing of strength in one extremity, or increased pathology in the other. MRI scans show muscles that are unaffected by the disease process often immediately adjacent to a profoundly damaged muscle [20], [21] despite the identical genetic state of the array in the myofibers of both muscles. In addition, the spectrum of disease severity suggests that epigenetic mechanisms are likely important for disease progression and pathogenesis. These observations, in addition to previous studies showing chromatin structural differences of pathogenic D4Z4 arrays, [7], [11] suggest that array chromatin structure and *DUX4* transcriptional regulation will be central themes for understanding the pathogenesis of FSHD.

Since sense and antisense transcription within other human repetitive elements has been previously described [3], [22], [23], we designed reporter constructs to determine if D4Z4 DNA elements participate in the initiation of bidirectional transcription. Gene expression cassettes that contain the non-coding region of D4Z4 flanked by fluorescent reporters were constructed so that transcription initiated in either direction could be easily detected and quantified. We show that initiation of transcription in the antisense direction is a hallmark of the D4Z4 promoter regardless of cell type but myoblasts and myotubes allow sporadic sense–strand transcription in a small percentage of cells. Furthermore we identify transcriptional elements that alter the balance of sense and antisense transcription and propose a model where antisense transcription may ultimately result in transcriptional silencing of the D4Z4 array.

## Results

### Reporter Constructs that Measure Transcriptional Activity of D4Z4 Sequences

**Figure 1:**
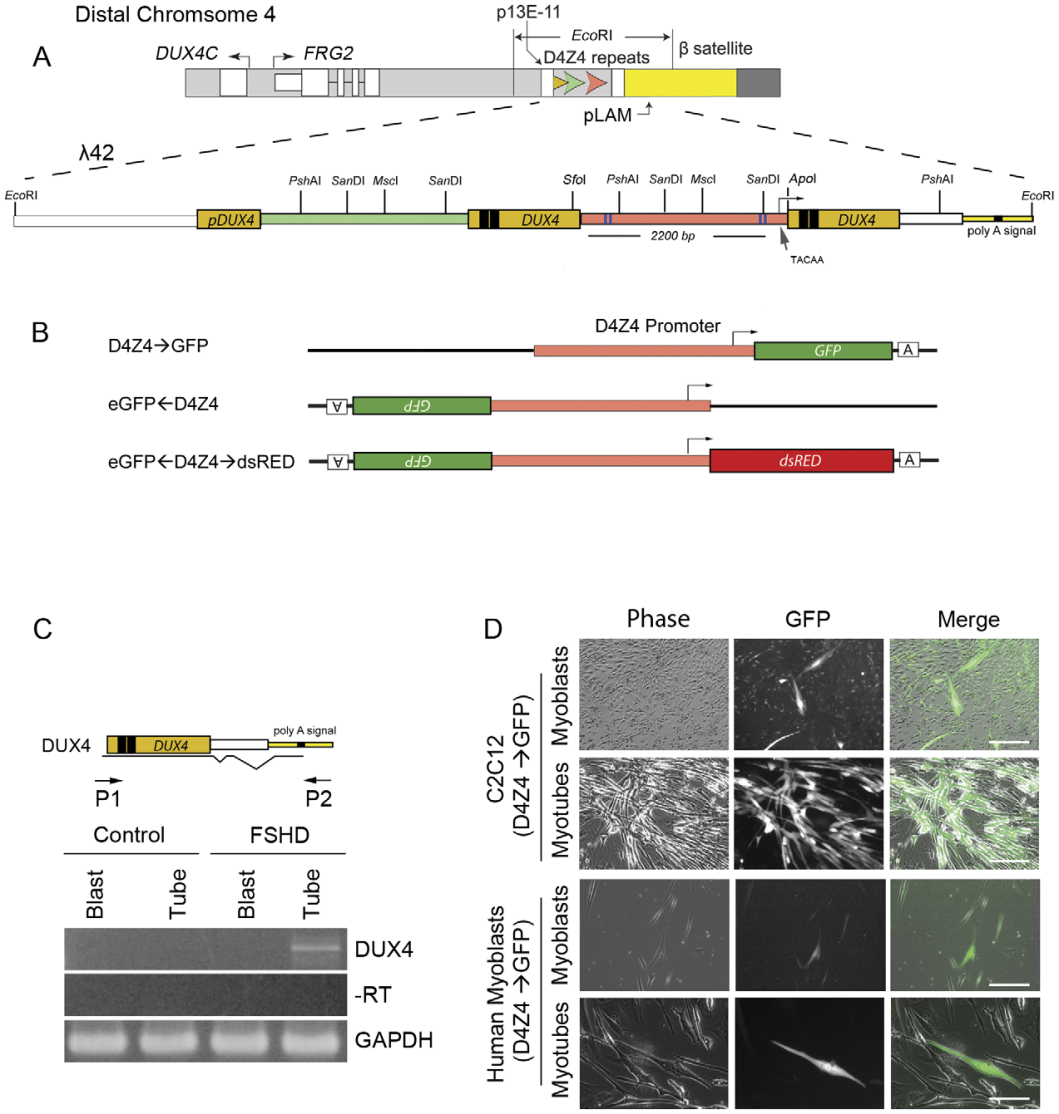
Reporter constructs and their activity in human and mouse myoblasts and myotubes. (A) The distal end of chromosome 4 is shown above a schematic of an *Eco*RI fragment cloned from an FSHD-affected individual (λ42). λ42 contains two full D4Z4 units (green and pink rectangles), and two partial D4Z4 units on either end. The *DUX4* open reading frame is shown as a yellow colored rectangle with homeoboxes shown as black boxes within each D4Z4 repeat. The approximate location of the TATA box (TACAA) and the transcription start site (bent arrow) are indicated. The restriction enzymes *Sfo*I and *Apo*I were used to clone the non-coding region. The location of previously identified miRNA fragments from D4Z4 are shown as blue lines [3] (B) The non-coding region of D4Z4 was isolated from the second repeat of λ42 and placed upstream or downstream of the indicated reporter genes (D4Z4→*eGFP*, sense promoter driving *eGFP*; *eGFP*←D4Z4, antisense promoter driving *eGFP*; *eGFP*←D4Z4→*dsRED*, *DUX4* promoter driving *dsRED* in the sense direction and *eGFP* in the antisense direction. (C) Control and FSHD Human myoblasts were differentiated into myotubes and assayed for *DUX4* expression by RT-PCR. Locations of primers 1 and 2 to detect *DUX4* transcripts are shown along with common splice sites within the *DUX4* transcripts. (D) Mouse and Human myoblasts transduced with a lentivirus vector encoding D4Z4→*eGFP* were sorted by flow cytometry, and expanded in culture. The cells were seeded at equal densities, switched to either myoblast proliferation medium or myotube differentiation medium for 72 hours, and imaged by fluorescence microscopy. Scale bars = 50 µm. Images were taken at the same time with the same exposure settings.

The non-coding region from the last full D4Z4 repeat of the previously cloned human disease locus, λ42 [24], was defined as the regulatory region and extends from the *Sfo*I site in the 3′ end of the first *DUX4* reading frame to the *Apo*I site downstream of the previously identified *DUX4* TATA box (TACAA), of the second *DUX4* gene (Fig. 1A) [1]. Expression constructs were developed to measure sense/forward transcription (D4Z4→*eGFP*), antisense/reverse transcription (*eGFP*←D4Z4), or bidirectional transcriptional regulation (*eGFP*←D4Z4→*dsRED*; Fig. 1B). Reporter expression was assayed in transfected cells, or after delivery by lentiviral transduction to prevent formation of head to tail concatamers that might secondarily affect promoter activity. Lentiviral vector constructs contained a 3′ LTR deletion such that all enhancer and promoter activity from the LTR sequences was removed upon integration [25] allowing us to attribute reporter transcription to the D4Z4 sequences present in these vectors.

### The D4Z4 Promoter Activates Transcription in Myoblasts and Myotubes

A notable regulatory feature of the *DUX4* promoter is the increase in transcription as human myoblasts begin to form myotubes in cell culture, shown previously by detection of transcripts and protein in human FSHD fibroblasts converted to myotubes by myoD expression and in myotube/myoblast pairs [4]. We repeated these findings (Figure 1C) and assayed expression in the same cells using GFP fluorescence to determine if the reporters shown in Figure 1B reflected these regulatory changes. Mouse C2C12 myoblasts and human myoblasts were transduced with lentivirus vectors containing the D4Z4 promoter and *eGFP* (Fig. 1, D4Z4→*eGFP*). GFP(+) cells were isolated by flow cytometry and seeded to separate dishes at equal density. When culture dishes reach 90% confluence the medium was changed to either myoblast proliferation medium or myotube differentiation medium, and fluorescence detected 72 hrs later. An increase in GFP fluorescence intensity was observed in multinucleated myotubes consistent with changes in *DUX4* transcript levels noted in the same cells (Figure 1D).

### Asymmetric, Bidirectional Transcription from D4Z4 in Myoblasts and Myotubes, but not Human ES Cells

**Figure 2:**
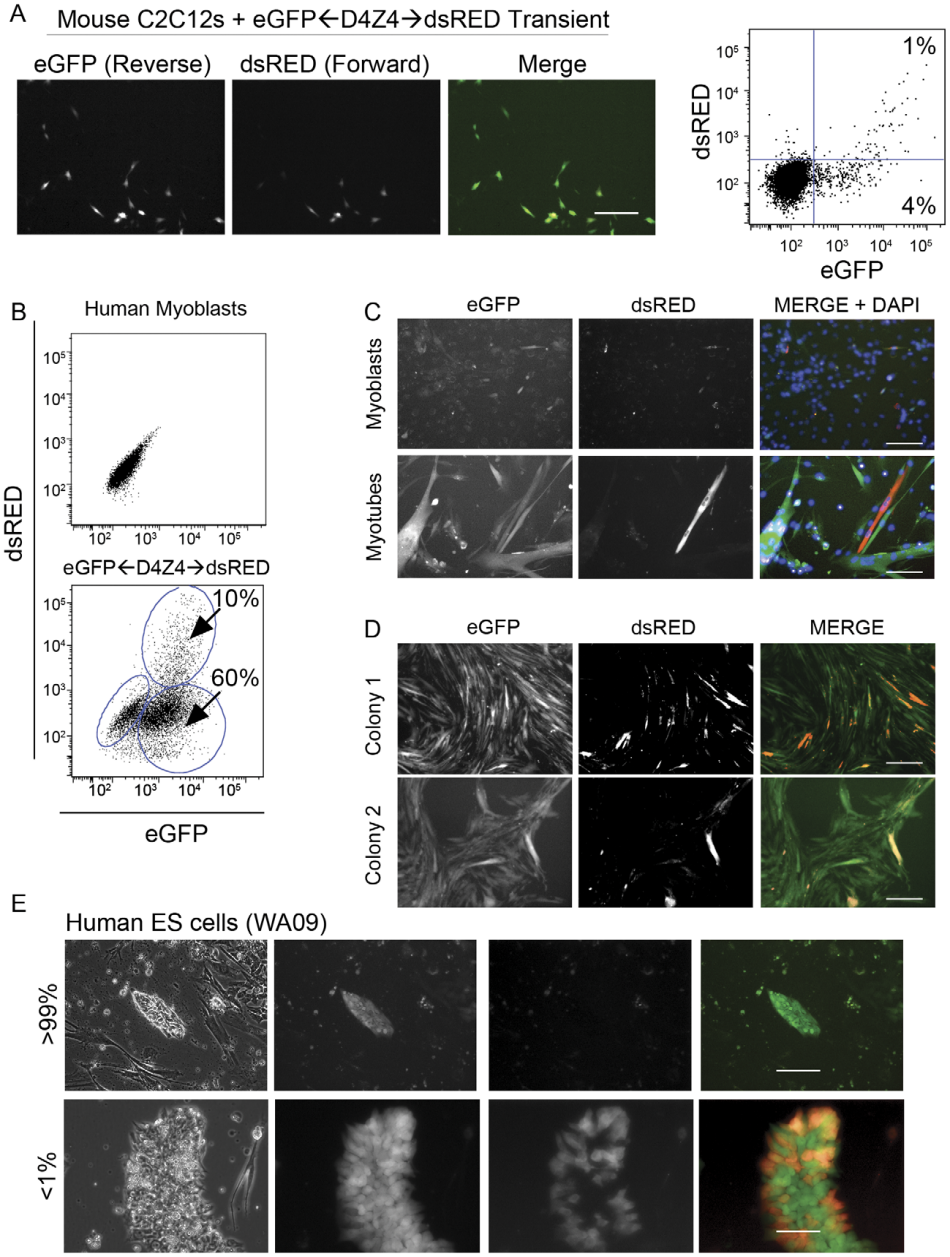
Transcriptional activity from D4Z4 in myoblasts, myotubes, and ES cells. (A) C2C12 cells were transfected with *eGFP*←D4Z4→*dsRED* and photographed using fluorescent microscopy 48 hours after transfection; scale bar = 50 µm. Fluorescence intensity measured using flow cytometry is shown as a scatter plot. Gates (blue lines) were determined with a control cell population that was not transfected. The numbers displayed in the quadrants represent the percent of the total population. (B) Human myoblasts were transduced with *eGFP*←D4Z4→*dsRED* and fluorescence intensity of cell populations was measured by flow cytometry. dsRED-GFP(+) cells and GFP(+) cells are circled in blue and the percentage of the total cell population indicated. (C) *eGFP*←D4Z4→*dsRED* infected human myoblasts were plated at equal densities and cultured in myoblast proliferation medium or myotube differentiation medium and photographed 72 hr later with fluorescence microscopy; scale bar = 50 µm. (D) *eGFP*←D4Z4→*dsRED* infected human myoblasts were plated at single cell density and two clones were imaged to demonstrate the assymetric reporter distribution. Arrows point to dsRED(+) cells to highlight that dsRED and GFP are proportionally regulated; scale bar = 50 µm. (E) Human ES cells (WA09) were infected with *eGFP*←D4Z4→*dsRED* lentivirus as a single cell suspension and grown for 24 hrs in the presence of Rho-associated coiled-coil forming protein serine/threonine kinase (ROCK) inhibitor Y27632 to prevent differentiation of the cells. After one week of culture, individual colonies were assayed for *eGFP* and *dsRED* expression by fluorescent microscopy and scored as either GFP(+) or dsRED-GFP(+). Top panel scale bar = 50 µm, bottom panel scale bar = 100 µm.

To investigate the possibility that bidirectional transcription is initiated from D4Z4 similar to other macrosatellite repeats [22], plasmids containing *eGFP*←D4Z4→*dsRED* were transfected into C2C12 cells and assayed for fluorescence by flow cytometry. Strikingly, a significantly larger fraction of cells expressed *eGFP* (antisense) compared to *dsRED* (sense) (Figure 2A). We quantified asymmetries of direction by dividing the number of dsRED(+) cells by the total fluorescent cell count (dsRED+GFP). Since 1% of the total cell population were dsRED(+), and 4% were GFP(+), directional asymmetry was 20% percent (Figure 2A). Although the reporters demonstrated significant asymmetries in transcriptional activity, both *GFP* and *dsRED* were usually expressed in the same cells. Very few cells could be identified that showed only *dsRED* expression suggesting that the asymmetric expression patterns are co-regulated.

Normal human myoblasts stably transduced by the *eGFP*←D4Z4→*dsRED* lentivirus vector showed a similar distribution of asymmetric bidirectional transcription (dsRED/(dsRED+GFP) = 14%) (Figure 2B). When the cells were differentiated into myotubes, the directional asymmetry was essentially unchanged but transcription from both reporters was upregulated (Figure 2C). To confirm that asymmetric expression pattern was not due to variable integration patterns of the lentivirus, the cells were seeded at clonal density and assayed for asymmetry by microscopy. Clonal populations were almost 100% GFP positive, and displayed consistent asymmetry in multiple clones (Figure 2D).

Since we previously noted *DUX4* transcripts in IPS cells from FSHD-affected individuals [4] we examined transcriptional directionality in human ES cells. dsRED positive cells (sense) were restricted to a subset of about 1 in 250 ES cell colonies, and these colonies contained a heterogeneous pattern of expression with forward transcription present in less than 0.5% of the colony. (Figure 2E). These data demonstrate that the D4Z4 promoter region initiates bidirectional transcription in myoblasts, but antisense transcription is favored in embryonic cell types. The previously detected sense transcripts are present in a small proportion of IPS cells consistent with the absence of detectable DUX4 protein in most cells in these cultures (data not shown).

### Transcription from D4Z4 is Dynamically Switching Directions

**Figure 3:**
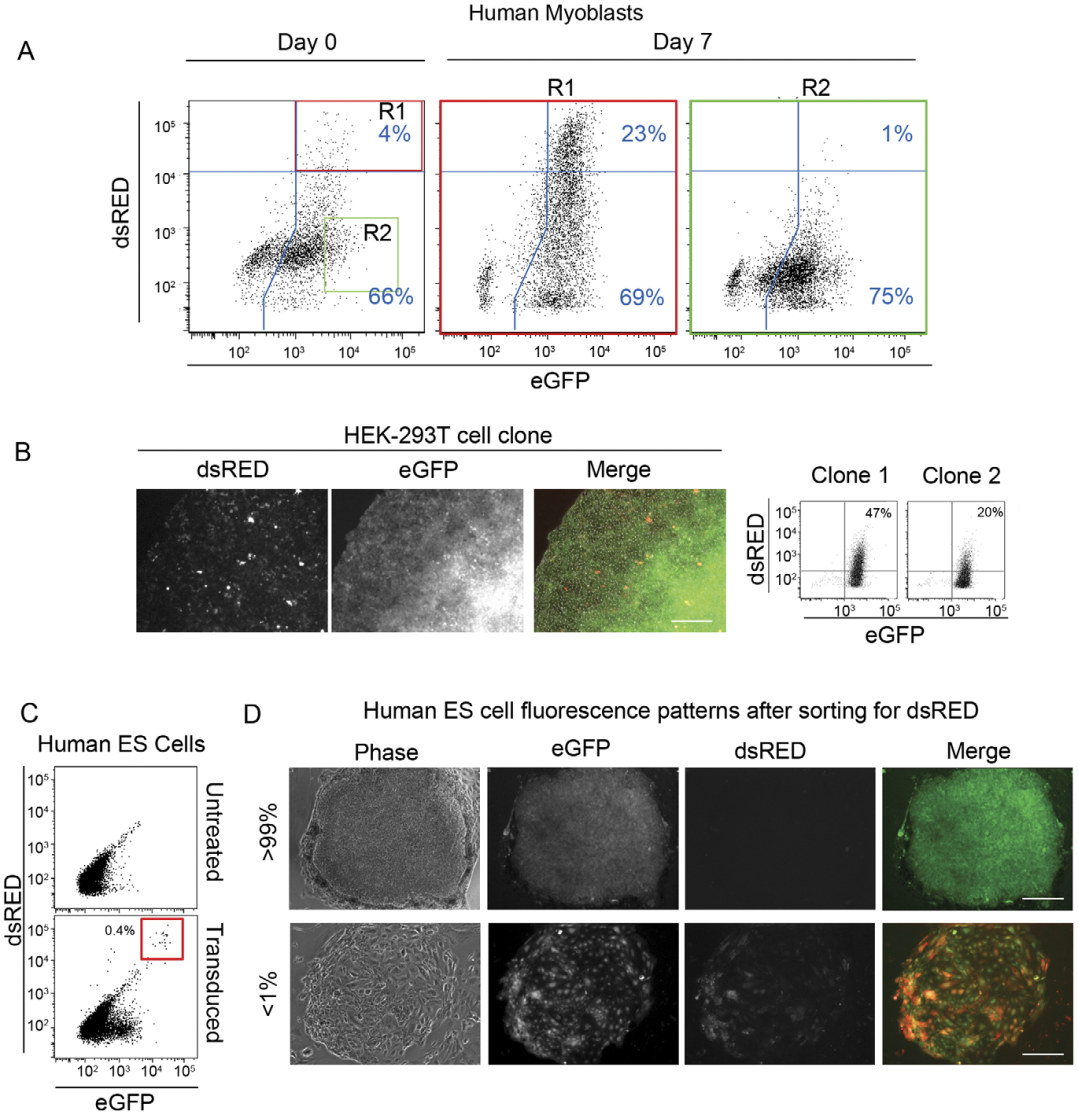
Transcription from D4Z4 is dynamically switching directions. (A) Human myoblasts were infected with *eGFP*←D4Z4→*dsRED*. After expression was observed (arbitrarily designated day 0) cells were sorted by flow cytometry to purify dsRED-GFP(+) cells (red box; R1) or GFP(+) cells (green box; R2). Gates were set one log above cut-off thresholds established in figure 1. dsRED(−)GFP(+) and GFP(+) cells were seeded into separate dishes and cultured until dishes were 85% of confluence. Cells were collected and analyzed by flow cytometry after 7 days of culture. The fluorescence intensity of cells collected from R1 or R2 are shown as a scatter plot and the percentage of cells meeting criteria for dsRED-GFP(+) or GFP(+) are labeled. (B) HEK-293T cells were infected with *eGFP*←D4Z4→*dsRED*, sorted by flow cytometry to obtain dsRED-GFP(+) cells and seeded to culture dishes as single cells. Single cell derived colonies were photographed at 20× magnification and clonally isolated for analysis by flow cytometry; scale bar = 50 µm. (C) WA09 Human ES cells were infected with *eGFP*←D4Z4→*dsRED* as a single cell suspension and cultured under conditions that prevent differentiation. Colonies containing red cells (∼1/250 GFP(+)) were isolated using glass cloning rings and transferred to new culture wells. To ensure clonal growth, the cells were subcloned four times by repeatedly isolating single-cell-derived colonies. The resulting population was less than 0.5% double positive for dsRED and GFP (red box). (D) Cells from (C) were sorted for dsRED-GFP(+) fluorescence and expanded as colonies that were scored for *dsRED* and *eGFP* expression by fluorescence microscopy; Scale bar = 50 µm.

The choice of transcriptional direction initiated from D4Z4 could be fixed, or change dynamically over time. To investigate the maintenance of a particular transcriptional direction in cells, human myoblasts positive for both dsRED (sense) and GFP (antisense), or GFP (antisense) only were isolated using flow cytometry. To carefully separate the double positive population from the GFP(+) population, gates were set one log above the minimal threshold for dsRED fluorescence. The 4% of cells that were double positive on day 0 gave rise to a cell population that contained 23% dsRED(+)GFP(+) cells. Similarly, the population of cells sorted for GFP produced cells that were red (sense); however the switch from antisense to sense occurred less readily (∼1% of the starting cell population) (Figure 3A). Comparable results were seen in clonal isolates of transduced HEK-293T cells (Figure 3B).

Transduction of ES cells with *eGFP*←D4Z4→*dsRED* gave rise to populations of cells that were mostly GFP(+) (antisense); whereas, dsRED GFP double positive cells were observed in about 1 in 250 GFP(+) colonies. Serial attempts to clone the cells expressing *dsRED* failed because isolated colonies always contained a mixture of red and green cells. Flow cytometry analysis of a clonal population containing red cells revealed 0.46% of the cells were dsRED(+); however, colonies derived from the sorted dsRED(+) cells were mostly GFP(+) (Figure 3D; top panel). dsRED(+) cells often appeared in the periphery of colonies where cells had begun to differentiate, or in colonies that had completely differentiated (Figure 3D; lower panel). Thus, bidirectional transcription from the D4Z4 promoter region is dynamic, and differentially regulated in myoblasts and embryonic stem cells.

### A Small Region of D4Z4 Influences the Balance of Sense and Antisense Transcription

**Figure 4:**
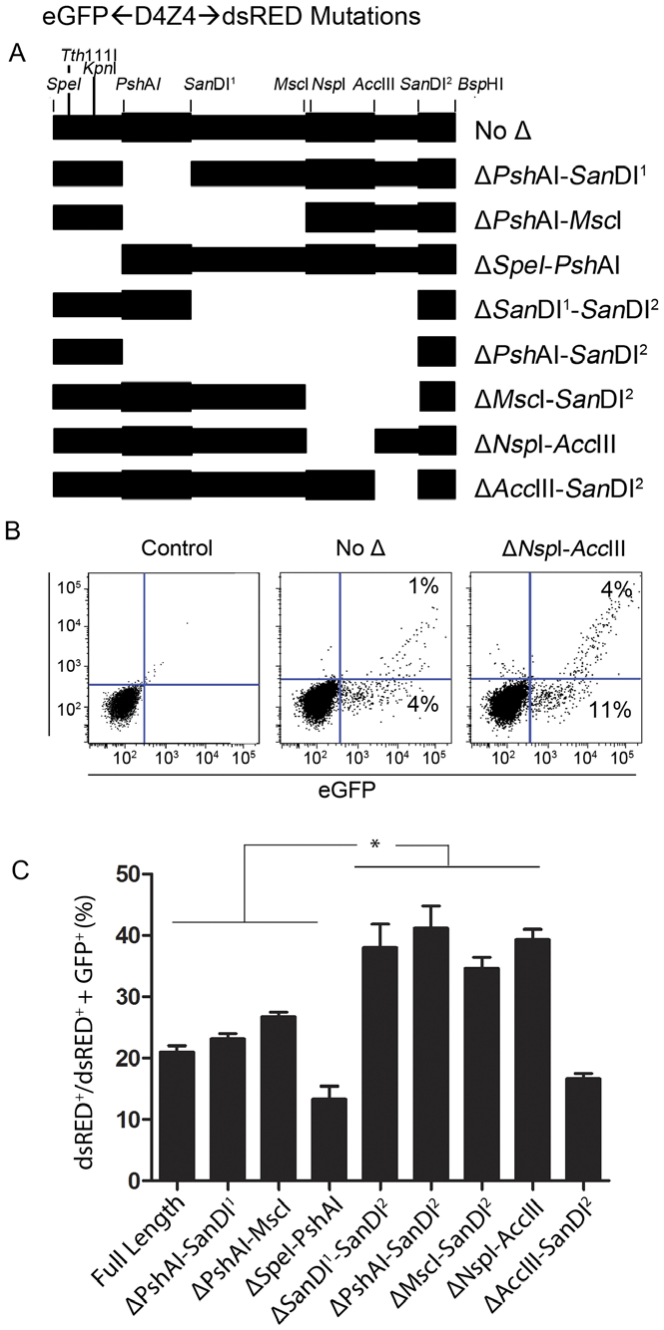
Mutational analysis of the non-coding region of D4Z4. (A) The non-coding region of D4Z4 with restriction enzyme sites used to construct various deletions is shown (no Δ) above the deletion series. Promoter regions involved in the various deletions are emphasized using alternating thickness of the rectangle and the name of the deletion is labeled at the right. (B) C2C12 cells were transfected with each mutant *eGFP*←D4Z4→*dsRED* and assayed for *dsRED* and *eGFP* expression by flow cytometry. The fluorescence intensity is plotted for the expression construct containing the most informative deletion (Δ*Nsp*I-*Acc*III) as well as mock transfected cells (Control) and cells transfected with the non-deleted vector (No Δ). (C) Quantification of the percentage of the total number of fluorescing cells that are dsRED-eGFP(+) for each mutation. Error bars = SD of the mean in three experiments that measured triplicate samples. * = p<0.05.

To identify D4Z4 sequences important for establishing transcriptional direction, mutations were created in the second full D4Z4 unit of the patient clone λ42 using restriction enzyme sites that divided the noncoding region of D4Z4 into 5 regions. The deletions were introduced into the expression construct (*eGFP*←D4Z4→*dsRED* (Fig. 4A)), and plasmids containing them were transfected into C2C12 cells and assayed by flow cytometry (Fig. 4B–C). To accommodate for differences in transfection efficiency, we calculated the percentage of dsRED(+) cells in the fluorescent cell population using the following equation: (dsRED(+)/(dsRED(+)+GFP(+))). Cells expressing reporters containing the Δ*Psh*AI-*San*DI^2^, Δ*San*DI^1^-*San*DI^2^, and *Msc*I-*San*DI^2^ deletions demonstrated a significant increase in percent of dsRED(+) cells when compared to the wild type promoter suggesting that the region between *Msc*I and *SanD*I^2^ was critical for inhibiting sense transcription (Fig. 4). Further analysis revealed that removal of the region between *Acc*III and *Nsp*I had the same effect as larger deletions and was effective at skewing the direction toward the sense strand in human myoblasts as well. Therefore this region of the promoter contains an important regulatory element that biases transcription initiation in the antisense direction and facilitates sense transcription when removed.

We performed a computational search to determine which transcription factors may be present in this segment and if their binding sites are unique to the region of the promoter. Of the binding sites identified, two were unique to the region between *Nsp*I and *Acc*III compared to the rest of the D4Z4 regulatory region (NF-E2, and TST1); however, neither NF-E2 or TST1 met criteria for further exploration as neither are expressed in skeletal muscle. We therefore focused on the transcriptional repressor, YY1, since its binding site was present four times within the region (Figure S1A) and was previously identified as a critical repressor of D4Z4 [26] and was recently found to play a role in regulation of DXZ4 [27]. Analysis of the *Nsp*I - *Acc*III fragment sequence against the core consensus sequence for YY1 [28] using Matrix Search for Transcription Factor Binding Sites (http://www.gene-regulation.com/cgi-bin/pub/programs/match/bin/match.cgi) confirmed the presence of the cluster of the putative YY1 binding sites within the sequence (Figure S1A). We performed electrophoretic mobility shift assays using nuclear extracts from 293 cells and *in vitro* translated YY1 protein with ^32^P-labeled double-stranded oligonucleotides corresponding to the putative YY1-binding sequences. Our results indicate that while both *in vitro* translated and endogenous YY1 specifically bound the control YY1 binding site, no binding to the D4Z4 sequences was detected (Figure S1B).

Removal of the previously identified TATA box [1], was accomplished by creating a deletion using the restriction enzyme *Bsp*HI. Plasmids encoding D4Z4→LacZ, D4Z4→LacZ (Δ*Psh*AI-*San*DI^2^), D4Z4→LacZ(Δ*San*DI^1^-*San*DI^2^), and D4Z4→LacZ (Δ*Bsp*HI), were transfected into C2C12 cells and β-galactosidase activity quantified relative to a co-transfected *Renilla* luciferase control vector (Figure S2). As we observed previously using flow cytometry, removal of the putative TATA box (TACAA) and associated transcription start sites resulted in a significant decrease in transcriptional activity highlighting the importance of these sequence elements for promoter activity.

### Antisense Transcripts Originate in a Cluster within a Second D4Z4 Promoter

**Figure 5:**
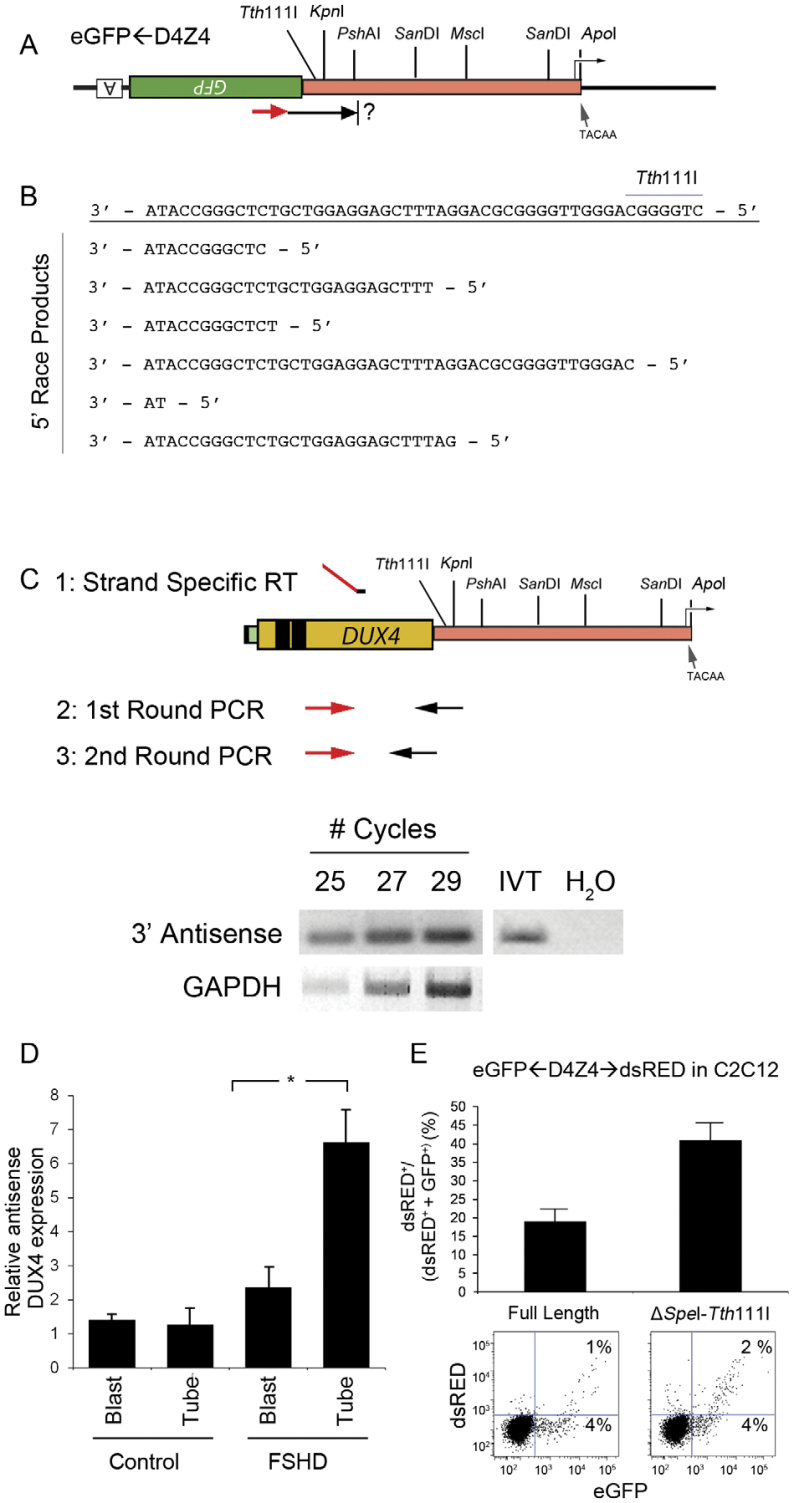
Antisense transcripts begin in a GC-rich region distal to the *DUX4* ORF. (A) 5′ RACE was performed on cells transduced with *eGFP*←D4Z4 lentivirus using primers based within the *eGFP* open reading frame. The *eGFP*←D4Z4 reporter construct is shown with the previously identified sense transcription start site indicated by a bent arrow. The *eGFP* gene and poly-adenylation signal (box with A) is shown upside down and backwards to indicate reporting transcription in the antisense direction. The primer used for cDNA synthesis with reverse transcriptase is shown as a red arrow with the location of the 5′ end of the antisense transcript indicated as a question mark. (B) Nucleotide sequence of the 5′ end of six separate isolates cloned from the 5′RACE reaction. The sequence is shown as a 3′-5′ strand to depict the antisense transcript. (C) Antisense-strand-specific RT-PCR strategy is diagramed. *DUX4* is indicated as yellow rectangle with black boxes showing the location of the homeobox motifs and is drawn adjacent to the antisense promoter region. *Tth*111I and *Kpn*I sites are shown pointing to their approximate location at the 5′ end of the antisense promoter. The strand specific RT primer contains a linker sequence (red line) that is complementary to primers used in subsequent PCR reactions (red arrows). Primers complementary to the antisense strand upstream of the *Tth*111I site are shown as black arrows. *In vitro* transcribed RNA was used as a positive control and H_2_O as a negative control. (D) Human control and FSHD myoblasts and myotubes were assayed by strand-specific RT-PCR for the antisense transcript, quantified by densitometry, and normalized to GAPDH transcripts in the same RNA preparations. N = 4. * p<0.05. (E) A plasmid containing *eGFP*←D4Z4(Δ*Spe*I-*Tth*111I)→*dsRED* containing a deletion of the start site cluster upstream of the restriction enzyme site *Tth*111I was transfected into C2C12 cells and fluorescence measured by flow cytometry. The graph shows the ratio of dsRED-GFP(+) cells to dsRED-GFP(+)+GFP(+) cells shown as a percentage. Error bars = SD of the mean of three experiments that measured duplicate samples. Lower panel: flow diagrams of the corresponding deletions.

Since antisense transcripts likely have important regulatory activities themselves, it is important to define their sequence and start sites. Cells were transduced with the lentivirus encoding *eGFP*←D4Z4 and RNA was prepared for use in 5′ RACE protocols. Using gene-specific primers within *GFP* (Figure 5A), a 33 bp region near the end of the *DUX4* open reading frame was identified that contained multiple transcription start sites (Figure 5B). Careful analysis of the sequence adjacent to these sites did not reveal a canonical TATA box suggesting that transcription is initiated from this promoter by a TATA-less mechanism. Consistent with these findings is the observation that other TATA-less promoters generally initiate transcription at a number of sites within a GC rich sequence [29]. With an understanding of where D4Z4 antisense transcripts originate, we utilized strand-specific RT-PCR to detect the endogenous antisense transcript using a gene specific RT primer homologous to the 3′ end of *DUX4*. The RT primer contained a linker sequence complementary to a forward primer used to achieve strand specificity in subsequent PCR reactions. Antisense transcripts were amplified and confirmed to originate from chromosome 4 by sequence identification of chromosome 4-specific SNPs. *In vitro* transcribed RNA was used as positive control (Figure 5C). Since we observed an increase in both sense and antisense transcription when myoblasts were differentiated into myotubes, we quantified antisense D4Z4 transcripts from control and FSHD-derived myoblasts and myotubes to determine if these transcripts were coregulated in patients, and if disease-specific differences might exist. As the reporter experiments would predict, we found that antisense transcription was upregulated in FSHD-derived myotubes (Figure 5D). The importance of this regulatory region was confirmed by demonstrating that deletion of the *Spe*I – *Tth*111I fragment containing these transcriptional start sites results in a shift toward sense transcription when fluorescence from the *eGFP*←D4Z4(Δ*Spe*I-*Tth*111I)→*dsRED* reporter is quantified in C2C12 cells (Figure 5E).

### The Distal Region of the Pathogenic D4Z4 Array Exclusively Promotes Antisense Transcription

**Figure 6:**
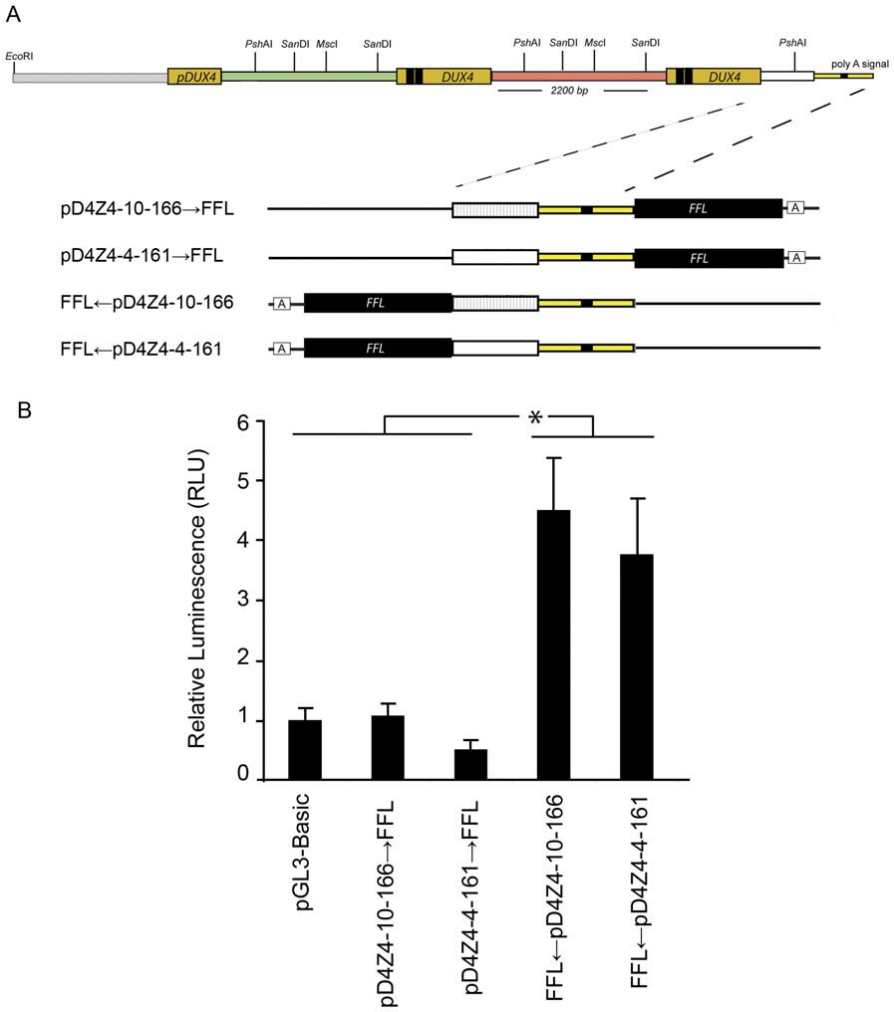
Antisense transcriptional activity from the last partial D4Z4 unit is unaffected by disease-associated haplotypes. (A) Schematic of distal unit of λ42. The white bar flanked by a dashed line represents the last partial unit of D4Z4 that leads into the region known as pLAM (yellow box) and contains the haplotype-specific poly adenylation signal for *DUX4* (black box). Firefly Luciferase (FFL) reporter constructs contained the distal partial subunit derived from a chromosome 4 that had a permissive haplotype in the SSLP region near P13E11 (161 bp length). Regions cloned from arrays with permissive haplotypes are shown as a white box (pD4Z4-4-161→*FFL* and *FFL*←pD4Z4-4-161) and those from the chromosome 10 non-permissive haplotype (sslp of 166 bp length) are shown by a white box with lines in orientations that report transcription in either sense or antisense directions (pD4Z4-10-166→FFL and FFL←pD4Z4-10-166). (B) A plasmid containing *FFL*←pLam and pLam→*FFL* was transfected into C2C12 cells and assayed for luminescence. pGL3 basic backbone was used as a negative control. Values were normalized to a co-transfected *Renilla* luciferase vector. Error bars = SD of the mean of six experiments that measured duplicate samples.

The most telomeric partial unit of the D4Z4 array and pLAM region contains sequence polymorphisms critical for *DUX4* expression and disease pathogenesis [2], [5]. Since this distal region lacks the start site required for transcription in the sense direction, we tested the hypothesis that transcription in the antisense direction might be haplotype dependent and contribute to disease pathogenesis (Figure 6A). A luciferase reporter system was used to compare the transcriptional activity of promoters containing permissive (4qA161) and non-permissive (10qA166) haplotypes in both directions. Transcription in the antisense direction was seen exclusively, and no differences were observed between constructs containing permissive or non-permissive haplotypes (Figure 6B).

## Discussion

Bidirectional transcription from a promoter can regulate gene expression in a number of ways that depend on how the genes flanking the promoter are oriented. DNA repair genes oriented head-to-head can be co-regulated by the same promoter and associated response elements [30]. Pervasive antisense transcription has also been described in yeast and plants where transcription through open reading frames or promoters can induce or suppress expression of the gene [31]. In vertebrates, bidirectional transcription initiated by promoters flanking open reading frames such as the p21 and p15 genes, generates antisense transcripts that participate in transcriptional regulation through an epigenetic mechanism [32]. Perhaps most relevant to D4Z4, the DXZ4 bidirectional promoter appears to establish the chromatin structure of the array in which it resides, resulting in heritable expression or silencing of transcription as a function of the surrounding chromatin context [22].

The production of DUX4 protein requires an open chromatin conformation [11] in addition to specific haplotypes involved in RNA stabilization and processing [2], [4], [5], [14]. When the number of units is less than 10, D4Z4 arrays adopt an open chromatin conformation despite efficient packaging into facultative heterochromatin of nearly identical arrays on the other chromosome 4 allele and on chromosome 10. Thus, the size of the array, or cis-acting transcripts coming from the array are likely signals for heterochromatin assembly. The signal appears to be produced independently of the D4Z4 haplotype because short arrays on chromosome 10, or short non-permissive arrays on chromosome 4 are also packaged as euchromatin [11] and contain a large percentage of unmethylated CpG dinucleotides [6]. Our results suggest that antisense transcription from promoter elements within the array is also independent of haplotype because no difference in transcription was observed whether the promoter was derived from a permissive (4qA161) or a non-permissive (10qA166) haplotype. Despite the fact that every myo-nucleus present in an FSHD-affected individual's muscle meets the known requirements for *DUX4* expression, a small percentage of cells appear to be producing DUX4 at any particular time [4]. Thus, our results suggest that transcriptional direction is also an important feature of DUX4 production and that embryonic and somatic cells are able to influence this directionality in different ways.

In addition to restraining DUX4 transcription, the head-to-tail orientation of *DUX4* and the opposing promoters described here results in the production of complementary sense and antisense RNAs. As with DXZ4, these RNAs may have a regulatory function and may affect transcription rates and initiation as well as the chromatin structure of D4Z4. It is possible that the production of siRNAs or miRNAs from hybridized sense and antisense RNA regulate the establishment or maintenance of chromatin structure *in trans*, as proposed previously [3], [32]. Indeed, three of the five previously identified D4Z4 miRNA-sized transcripts align within the *DUX4* regulatory region (Figure 1A) and despite the fact that they do not overlap directly with the region identified by our deletion analysis their affect may well extend beyond the specific hybridizing region. Alternatively, the production of long non-coding RNAs that hybridize to genomic DNA may provide signals to recruit silencing factors *in cis*. This would be consistent with the observation that euchromatin is restricted to the truncated alleles in FSHD [33], [34]. Last, the observation that bidirectional transcription is altered in human embryonic stem cells and that *DUX4* transcripts are prevalent in germline cells allows for the possibility that piRNAs could play a role in regulation of *DUX4* transcription and potentially establish epigenetic signatures early in development that may be important for productive *DUX4* transcription in mature muscle. [35].

A current model of FSHD pathogenesis postulates that loss of heterochromatin at D4Z4 and the presence of a specific haplotype on chromosome 4 are necessary but not sufficient for *DUX4* expression. Our study adds to this model by demonstrating that the choice of transcriptional direction from each D4Z4 unit is an additional requirement for DUX4 production. Influencing this choice pharmacologically will likely be an important strategy for disease treatment.

## Materials and Methods

### Cell lines and Reagents

C2C12 mouse myoblasts (a gift from Dr. Stephen Tapscott [4]) and HEK-293T cells [36] were cultured in Dulbecco's Minimal Essential Medium (DMEM; Invitrogen, Carlsbad, CA; www.invitrogen.com) supplemented with 20% fetal bovine serum (FBS; Thermo Scientific (Hyclone); Rockford, IL; www.thermoscientific.com) and 50 U/50 µg penicillin/streptomycin (Pen-Strep; Invitrogen). Differentiation into myotubes was induced by culturing the cells in DMEM supplemented with 1% equine serum (Thermo Scientific (Hyclone)).

Primary human myoblasts were obtained through the Fields Center at the University of Rochester cell bank (http://www.urmc.rochester.edu/fields-center/protocols/myoblast-cell-cultures.cfm). Myoblasts were grown on dishes coated with .01% Calf skin collagen (Sigma Aldrich; #C9791) in F10 medium (Invitrogen) supplemented with 20% FBS, Pen-Strep, 10 ng bFGF (Invitrogen), and 1 µM dexamethasone (Sigma Aldrich; St. Louis, MI, www.sigmaldrich.com) [4]. Differentiation was induced using F10 medium supplemented with 1% equine serum and ITS supplement (insulin 0.1%, 0.000067% sodium selenite, 0.055% transferrin; Invitrogen; #51300-044).

### Ethics Statement

Control and FSHD cell lines were obtained from patients using consents approved by the IRB at the University of Rochester (approval numbers 12146 and 22880). Study subjects gave written informed consent for the use of their cells in these studies.

### DNA Cloning and Lentivirus Construction

The non-coding region of D4Z4 was isolated as an *Sfo*I-*Apo*I restriction fragment from the second full D4Z4 unit of the FSHD disease locus, λ42, and sublcloned into pBluescript. Virus vector preparations were generated by co-transfection of plasmids containing components of third generation lentivirus into HEK-293T cells using using polyethylimine (PEI) (3 ug DNA: 1 ug PEI (1 uL at 1 ug/uL)). For studies investigating antisense transcription from the distal repeat, D4Z4-Distal/pLam regions were PCR-amplified from pLambda42, pCS2-DUX4-4qA161, and pCS2-DUX4-10q.8 (contains 12 point mutations corresponding to 10q166 haplotype). *Nhe*I and *Hind*III sites were introduced at the 5′end of each sequence and *Xho*I site - at the 3′end. Forward (sense orientation) constructs were produced by sub-cloning D4Z4-Distal/pLam PCR fragments into *Nhe*I and *Xho*I sites of pGL3 basic (Promega). Reverse (anti-sense orientation) constructs were produced by sub-cloning D4Z4-Distal/pLam PCR fragments into *Xho*I and *Hind*III sites of pGL3 basic.

### Stem Cell Culture

Human ES cells (WA09 established line [37]) were cultured in DMEM:F12 (1∶1) with 3.151 g/L glucose, supplemented with L-Glutamine (Invitrogen), non-essential amino acids (10 mM Invitrogen, # 11140-076), sodium pyruvate (Invitrogen, 100 mM # 11360-070), 20% knockout serum replacer (Invitrogen, # 10828010), 1 mM 2-mercaptoethanol (Sigma Aldrich), and 5 ng/ml basic fibroblast growth factor (Invitrogen). Human ES cells were generally cultured on 0.1% gelatin coated dishes containing irradiated mouse embryo fibroblasts at a density of 1.3×10^4^ cells/cm^2^. When Human ES cells were used as a source of RNA, DNA, or protein they were cultured on matrigel (1∶60 dilution, BD biosciences, #356234) coated dishes in TeSR-2 medium (Stem Cell Technologies) and passaged as single cell suspensions using TrypLE recombinant trypsin and seeded at 1000 cells/cm^2^ in human ES cell medium containing 5 µM Y-27632 dihydrochloride and 0.5 µM Thiazovivin (Tocris Bioscience; Bristol UK).

### Semi-Quantitative RT-PCR

RNA was isolated by Trizol extraction according to the manufacturers protocol (Invitrogen), except that the RNA precipitate was resuspended in 100 µL H_2_O and then purified further using an RNA binding column according to the manufacturer's instructions (RNeasy Mini Kit, Qiagen). RNA was treated with 5 U DNase I for 15 mins and heat inactivated in the presence of 0.5 mM EDTA at 75°C for 10 mins. 1 µg RNA was converted to DNA by reverse transcription using Oligo dT primers and the Superscript III 1st strand cDNA synthesis kit. The RT reaction was incubated first at 65°C for 5 min, followed by 45°C for 45 min, 50°C for 15 min, and 55°C for 15 min, and 75°C for 15 min in a total of 50 µl. PCR for DUX4 was performed using the PCRX enhancer system for GC rich sequences (Invitrogen) and Taq polymerase (New England Biolabs). Cycling conditions: 35 cycles of 94°C×30 s, 55°C×30 s, 68°C×120 s. Primer Sequences: 652: CTCCCGACACCCTCGGACAGCAC; 588: CCAGGAGATGTAACTCTAATCCAGGTTTGC.

### Strand-Specific Quantitative RT-PCR

Strand-specific RT-PCR was achieved by performing the RT reaction as outlined above using 2 pmol of a gene specific primer containing a linker for subsequent amplification. RT primer: 5′-CGACTGGAGCACGAGGACACTGAccctgcgcggtggcacagcctg-3′ (linker sequence in caps). First round PCR primers: 5′-CGACTGGAGCACGAGGACACTGA-3′ and 5′-GGCCTCCGTTTCTAGGAGAGGTTGCGCCTGCTG-3′. Second round PCR: was performed using 5′-CGACTGGAGCACGAGGACACTGA-3′ and 5′-CCCCGGCCCCAGCCCCACCACGGACTCCC-3′ for the amplification reaction. The first PCR amplification occurred over 20 cycles, and a linear range was determined for the second amplification reaction. D4Z4 antisense expression was normalized to GAPDH expression and data presented as +/− standard error of the mean.

### Transfection

Transfection of plasmids into C2C12 myoblasts was performed using the GeneJuice transfection reagent (Novagen). DNA (0.5 µg) was diluted into 500 µL serum-free medium and incubated for 5 min at 23°C. 1.5 µL GeneJuice Reagent was added to the mixture, incubated 15 min and added directly to cultures containing DMEM+10% FBS. Transfection of plasmids into human myoblasts was performed using polyethylimine (PEI). DNA (2 µg) was diluted in 40 µL DMEM, and incubated with 6 µL PEI for 15 min. Cells were seeded at a density of 5000 cells/cm^2^ prior to transfection and assayed 48 hrs later.

### Flow Cytometry

Cells were analyzed on a BD FacsCanto II (BD Biosciences, Sparks MD, www.bdbiosciences.com). Cells were sorted using a BD FacsAria II. Analysis of data was performed using FloJo software (Tree Star Inc. Ashland, OR, www.flowjo.com).

### Luciferase Assays

Detection of firefly and *Renilla* Luciferase was performed using the Promega Dual Glo Luciferase Kit (Promega, Madison WI, www.promega.com), according to manufacturer instructions, and assayed using the Synergy 2 Multi-Mode microplate reader (Biotek, Winooski VT,www.biotek.com).

### Transcription Factor Binding Site Analysis

The sequence between the restriction enzyme sites *Nsp*I and *Acc*III from the D4Z4 regulatory region was analyzed for candidate transcription factor binding sites using MATCH, Matrix Search for Transcription Factor Binding Sites (http://www.gene-regulation.com/cgi-bin/pub/programs/match/bin/match.cgi). The presence of candidate binding sites was compared between the target fragment and the rest of the D4Z4 regulatory region. Transcription factor binding sites that were uniquely present in the *Nsp*I to *Acc*III region were considered.

### YY1 Electrophoretic Mobility Shift Assay

YY1 protein was *in vitro* translated using TnT Quick Coupled Reticulocyte Lysate Transcription/Translation System (Promega) from the plasmid, pBS-YY1, containing the YY1 cDNA sequence under T7 promoter (Received from Dr. Austen [38]). Nuclear extracts isolation and EMSA were performed as described previously [39].

## Supporting Information

Figure S1 **Analysis of YY1 binding to the** ***Nsp*** **I –** ***Acc*** **III fragment of the D4Z4 regulatory region.** (A) MATCH, Matrix Search for Transcription Factor Binding Sites (http://www.gene-regulation.com/cgi-bin/pub/programs/match/bin/match.cgi), identified a cluster of 4 putative YY1 binding sites (D4Z4 1–4) within the *Nsp*I – *Acc*III fragment of the D4Z4 regulatory region, each with a matched consensus YY1 binding site (highlighted in green). Sequences of the previously characterized YY1 binding site from the NCBI Reference Sequence NM_205332.4 (YY1 control) and the mutated YY1 binding site (YY1 mutant) are also indicated. (B) Electrophoretic mobility shift assays (EMSA) with ^32^ P-labeled double-stranded oligos shown in (A) and *in vitro* translated YY1 protein (Ivt YY1) and lysate control (No protein) or endogenous YY1 from nuclear extracts of 293 cells (NE). The results show that both *in vitro* translated and endogenous YY1 protein specifically interacted with the control YY1 binding site (YY1 con), but not the D4Z4 sequences. YY1 interaction with the control YY1 site was abolished by the mutation of the site (YY1 mut). Addition of anti-YY1antibody (C-20, Santa Cruz, Biotechnology) resulted in super-shift. Shifted YY1-DNA complexes are indicated as YY1. Note that *in vitro* translated YY1 was shifted as two bands with the lower molecular weight band corresponding to the YY1 protein free of post-translational modifications; whereas endogenous YY1 shifted as a single band corresponding to the higher-molecular weight band in IVT YY1 lanes.
(TIF)

Figure S2 **The previously identified TATA box TACAA, is required for sense transcription from D4Z4 reporter constructs.** A schematic diagram of the D4Z4→LacZ showing the location of restriction enzyme sites used to generate deletions. A sequence is provided to show the relationship between the *Bsp*HI site (red), TACAA TATA box (green) and ATG start codon of LacZ (blue). As in Figure 4, deletions Δ*Psh*AI-*San*DI^2^ and Δ*San*DI^1^-*San*DI^2^ showed increased production of sense transcriptional activity. The TACAA site was removed by deleting the region between *Bsp*HI and the ATG start site (Δ*Bsp*HI-ATG).
(TIF)
