# Supplementary figures and images for: Asymmetric Bidirectional Transcription from the FSHD-Causing D4Z4 Array Modulates DUX4 Production
Source: PLoS One. 2012 Apr 20;7(4):e35532. doi: 10.1371/journal.pone.0035532 (PMC3334996; doi:10.1371/journal.pone.0035532)

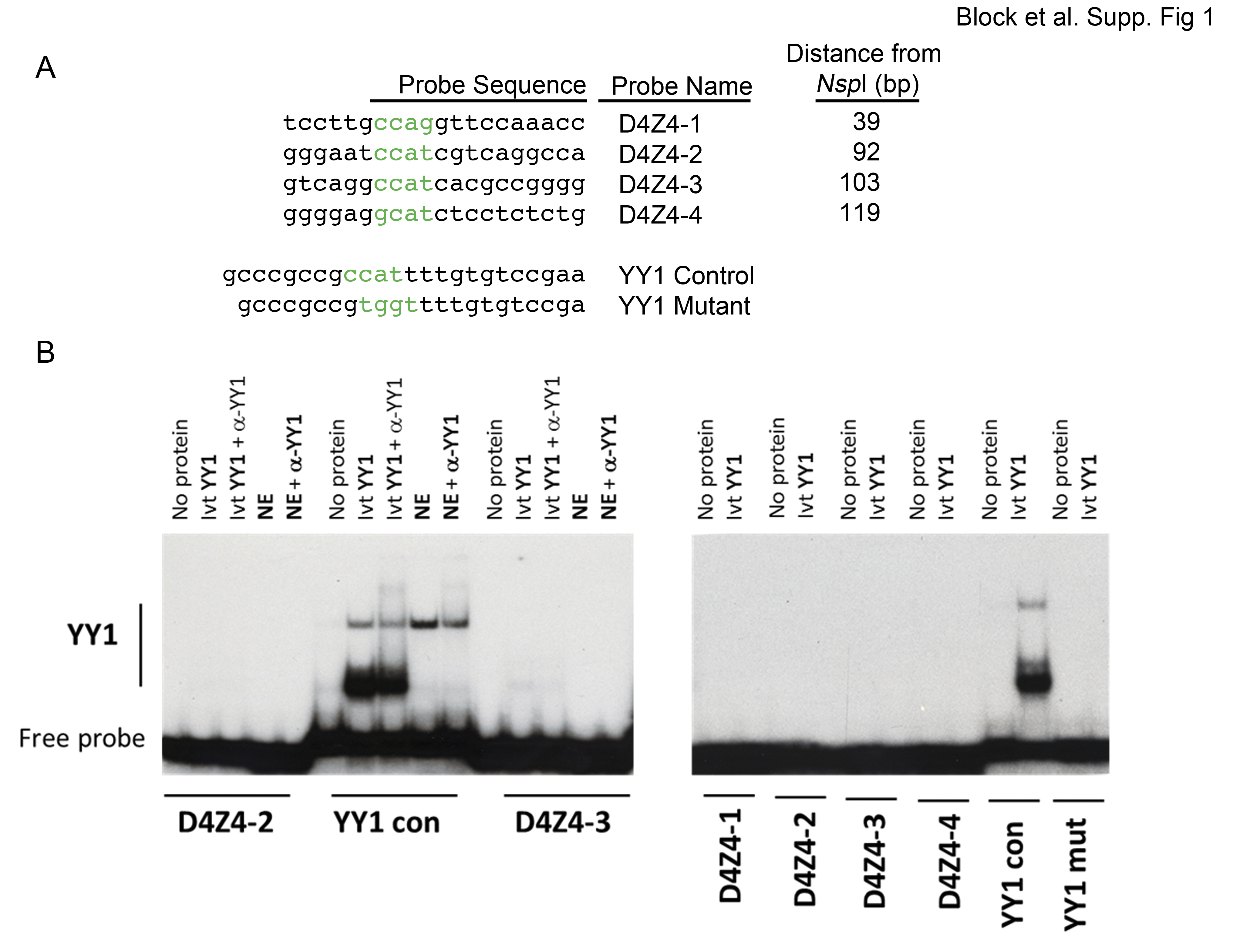

Supplement: Figure S1 — Analysis of YY1 binding to the Nsp I – Acc III fragment of the D4Z4 regulatory region. (A) MATCH, Matrix Search for Transcription Factor Binding Sites (http://www.gene-regulation.com/cgi-bin/pub/programs/match/bin/match.cgi), identified a cluster of 4 putative YY1 binding sites (D4Z4 1–4) within the NspI – AccIII fragment of the D4Z4 regulatory region, each with a matched consensus YY1 binding site (highlighted in green). Sequences of the previously characterized YY1 binding site from the NCBI Reference Sequence NM_205332.4 (YY1 control) and the mutated YY1 binding site (YY1 mutant) are also indicated. (B) Electrophoretic mobility shift assays (EMSA) with 32 P-labeled double-stranded oligos shown in (A) and in vitro translated YY1 protein (Ivt YY1) and lysate control (No protein) or endogenous YY1 from nuclear extracts of 293 cells (NE). The results show that both in vitro translated and endogenous YY1 protein specifically interacted with the control YY1 binding site (YY1 con), but not the D4Z4 sequences. YY1 interaction with the control YY1 site was abolished by the mutation of the site (YY1 mut). Addition of anti-YY1antibody (C-20, Santa Cruz, Biotechnology) resulted in super-shift. Shifted YY1-DNA complexes are indicated as YY1. Note that in vitro translated YY1 was shifted as two bands with the lower molecular weight band corresponding to the YY1 protein free of post-translational modifications; whereas endogenous YY1 shifted as a single band corresponding to the higher-molecular weight band in IVT YY1 lanes. (TIF) [file pone.0035532.s001.tif]

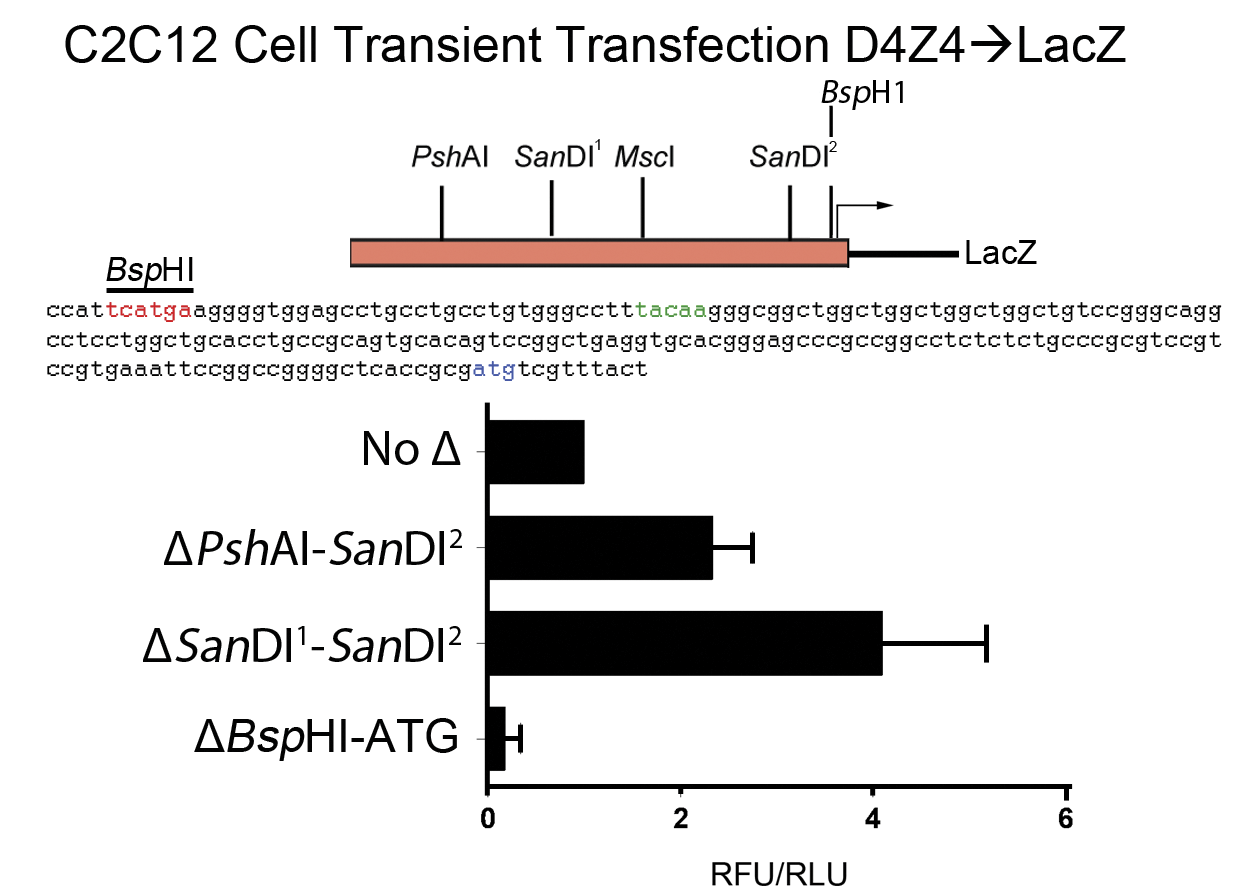

Supplement: Figure S2 — The previously identified TATA box TACAA, is required for sense transcription from D4Z4 reporter constructs. A schematic diagram of the D4Z4→LacZ showing the location of restriction enzyme sites used to generate deletions. A sequence is provided to show the relationship between the BspHI site (red), TACAA TATA box (green) and ATG start codon of LacZ (blue). As in Figure 4, deletions ΔPshAI-SanDI2 and ΔSanDI1-SanDI2 showed increased production of sense transcriptional activity. The TACAA site was removed by deleting the region between BspHI and the ATG start site (ΔBspHI-ATG). (TIF) [file pone.0035532.s002.tif]
